# A novel sphincter-sparing procedure for seton removal in complex perianal fistulas: the FiLaFlap technique

**DOI:** 10.1007/s10151-025-03271-8

**Published:** 2025-12-24

**Authors:** M. Cricrì, A. Miele, F. P. Tropeano, A. Zoretti, G. D. De Palma, G. Luglio

**Affiliations:** 1 Department of Clinical Medicine and Surgery, Endoscopic Surgery Unit, University of Naples “Federico II”, Naples, Italy; 2 Department of Advanced Biomedical Sciences, Endoscopic Surgery Unit, University of Naples “Federico II”, Naples, Italy; 3 Department of Public Health, Endoscopic Surgery Unit, University of Naples “Federico II”, Naples, Italy

**Keywords:** Perianal fistula, FiLac, Sphincter-sparing techniques

## Abstract

**Background:**

Complex perianal fistulas present a significant surgical challenge due to high recurrence rates and the need to preserve sphincter function. Fistula-tract Laser Closure (FiLaC™) is a minimally invasive technique that offers promising results, but incomplete closure of the internal opening remains a major cause of recurrence. To improve outcomes, we propose the FiLaFlap technique, which combines FiLaC™ with a mucosal advancement flap to enhance fistula healing.

**Methods:**

We conducted a retrospective study of patients who underwent seton removal with the FiLaFlap procedure between January 2023 and September 2024. Postoperative data, including pain levels, complications, continence status, and follow-up outcomes, were collected prospectively. Patients underwent clinical evaluations and 3D endoanal ultrasound assessments at predefined intervals.

**Results:**

A total of 24 patients (58.3% male, mean age 39.4 ± 12.4 years) were included. The mean time between seton placement and FiLaFlap was 5.95 ± 1.78 months. Postoperative pain was low (VAS 2.20 ± 0.97), and no major complications occurred. At 6 months, 91.6% of patients achieved clinical remission, while 83.3% had ultrasound-confirmed healing. Only one patient reported minor incontinence.

**Conclusion:**

The FiLaFlap technique appears to be a safe and potentially effective sphincter-preserving strategy for complex perianal fistulas, demonstrating high remission rates with minimal morbidity. Further studies with larger cohorts and extended follow-up are needed to validate these preliminary findings.

## Introduction

Complex perianal fistulas are a challenging entity in colorectal surgery, characterized by their intricate anatomy and association with high recurrence rates and significant morbidity. Surgical management aims to eradicate the fistula while preserving anal sphincter function and preventing postoperative complications such as fecal incontinence. Traditional approaches, including fistulotomy and fistulectomy, often involve significant risks when treating complex fistulas, especially those associated with extensive sphincter involvement [1, 2].

Fistula-tract Laser Closure (FiLaC™) has emerged as a promising minimally invasive option. This technique utilizes a radial laser probe to deliver controlled photothermal energy, inducing fibrosis and closure of the fistula tract. FiLaC™ is particularly attractive owing to its sphincter-preserving nature, making it a suitable option for complex fistulas. However, reported success rates for FiLaC™ vary, with primary healing rates ranging from 60% to 80% depending on the fistula’s etiology and anatomy [3–5].

To improve outcomes, FiLaC™ is increasingly being combined with other surgical techniques. One such combination is the mucosal advancement flap, which involves mobilizing and advancing a tension-free mucosal flap to cover the internal opening of the fistula. This technique complements FiLaC™ by addressing one of the main sources of recurrence: incomplete closure of the internal opening. Preliminary studies suggest that combining FiLaC™ with an advancement flap significantly enhances healing rates compared to FiLaC™ alone, with success rates reported as high as 90% in some series [6].

The purpose of this study is to evaluate the outcomes of the combined FiLaC™ and mucosal advancement flap (FiLaFlap) procedure in the treatment of complex perianal fistulas. Specifically, we aim to assess the rates of clinical and ultrasound-confirmed remission and provide insights into the safety and feasibility of this approach. By addressing the limitations of standalone techniques, this study seeks to contribute to the development of optimized, sphincter-preserving strategies for complex fistula management.

## Methods

### Patient population

All patients who underwent seton removal with the FiLaC plus mucosal advancement flap (FiLaFlap) procedure for complex perianal fistulas at our institution between January 2023 and September 2024 were retrospectively identified using a prospectively maintained database.

During the study period, all consecutive patients presenting with complex perianal fistulas—defined according to American Society of Colon and Rectal Surgeons (ASCRS) guidelines—previously treated with fistulectomy and seton placement were managed with the FiLaFlap procedure.

Complex perianal fistulas were defined according to the ASCRS guidelines as transphincteric fistulas involving more than 30% of the external sphincter, suprasphincteric, extrasphincteric, or horseshoe fistulas, as well as anal fistulas associated with inflammatory bowel disease (IBD), radiation, malignancy, preexisting fecal incontinence, chronic diarrhea, or anterior fistulas in women.

The study received approval from the local ethics committee.

### Surgical procedure

All surgeries were performed by a single consultant colorectal surgeon. Prior to the FiLaFlap procedure, all patients had undergone fistulectomy with seton placement at the same institution and by the same surgeon.

The FiLaFlap procedure was performed after an average interval of 6 months following seton placement, allowing for adequate tract maturation and stabilization of inflammation, in accordance with our institutional protocol.

Patients were placed in the standard lithotomy position and operated on under spinal anesthesia. First, the fistula tract was curetted to remove granulation tissue and debris. After the seton removal, a laser fiber probe was introduced into the tract up to the internal opening, and laser energy (continuous wave mode, 12 W, 1470 nm) was applied from the internal to the external orifice. A trapezoid-shaped, tension-free mucosal advancement flap was then created, advanced to cover the internal opening, and sutured in place using interrupted absorbable stitches.

### Data collection

Demographic data and postoperative outcomes were prospectively recorded for all patients.

Demographic data included age, sex, smoking status, etiology of the fistula (IBD or cryptogenic), ASA (American Society of Anesthesiologists) score, fistula type according to Park’s classification, and the time interval between seton placement and removal with the FiLaFlap technique.

Early postoperative pain was measured using the Visual Analog Scale (VAS).

All patients underwent clinical evaluation and 3D endoanal ultrasound prior to surgery, and were followed up with regular outpatient visits (weekly during the first month, then every 2 to 3 weeks during the second and third months postoperatively) and with 3D endoanal ultrasound performed approximately 6 months after surgery.

**Fig. 1:**
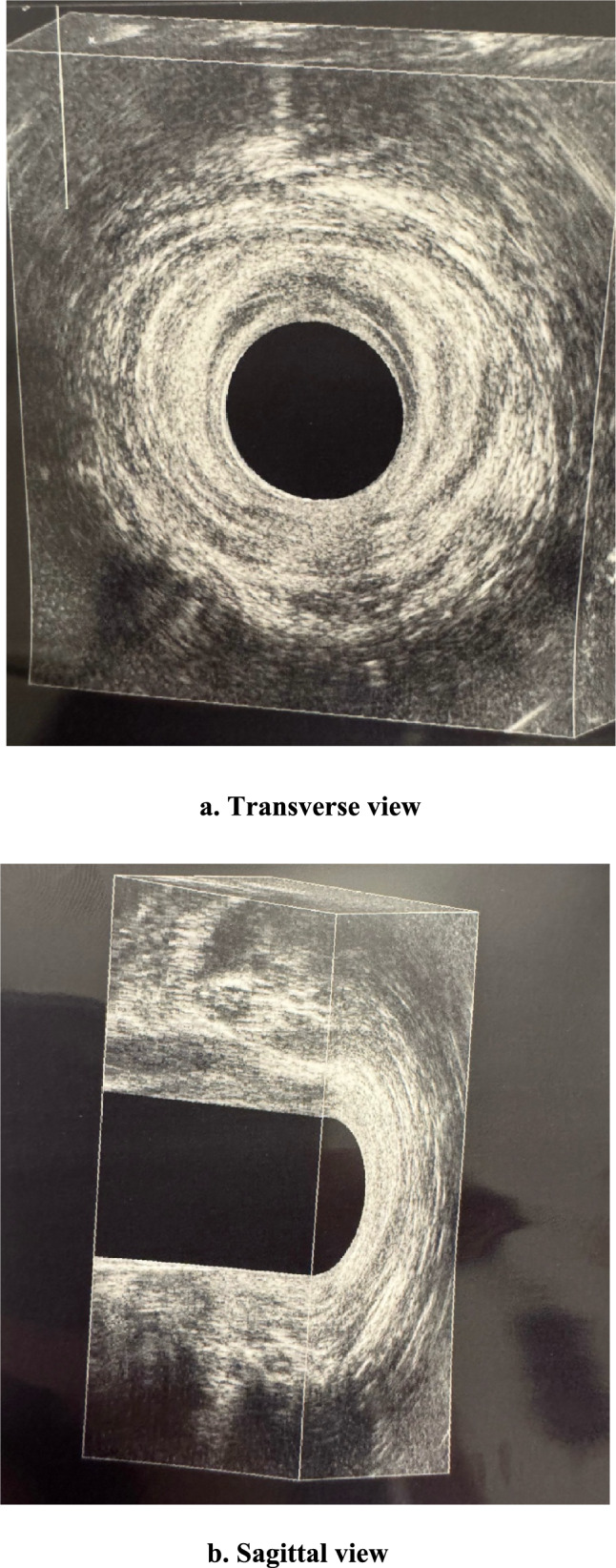
A 6-month endoanal ultrasound showing the presence of homogeneous tissue of intermediate echogenicity, without active fistulas or inflammatory collections, located posteriorly (6 o’clock). **a** Transverse view. **b** Sagittal view

Clinical remission was defined as the absence of pus or mucous discharge from any external opening or the anus upon gentle finger pressure. Ultrasound-confirmed remission was defined as the replacement of the previous fistulous tract with homogeneous tissue of intermediate echogenicity, indicative of scar replacement, in the absence of active fistulous tracts or inflammatory collections on follow-up 3D endoanal ultrasound (Fig. 1).

The presence of incontinence was also assessed and classified as minor incontinence (soiling) or major incontinence (fecal loss).

## Results

A total of 24 patients (58.3% male, mean age 39.4 ± 12.4 years) underwent the FiLaFlap procedure during the study period. Patients’ characteristics are showed in Table 1.

**Table 1:** Features of the study population

	Patients (*n* = 24)
Mean age (± SD)	39.4 ± 12.4
Male/female	14:10
Fistula type	
Crohn’s disease fistula, *n* (%)	9 (37.5%)
Recurrent anal fistula, *n* (%)	3 (12.5%)
Double-branch fistula, *n* (%)	2 (8.3%)
High transphincteric, *n* (%)	22 (91.6%)
Time to FiLaFlap procedure, months	5.95 ± 1.78

Among them, nine patients (37.5%) had Crohn’s disease, three (12.5%) had a history of recurrent anal fistula, and two (8.3%) had a double-branch fistula. The majority of patients (22, 91.6%) presented with high transphincteric fistulas based on intraoperative and ultrasound findings, while the remaining two (8.3%) were female patients with anterior anovestibular fistulas.

All patients with Crohn’s disease were in clinical remission at the time of surgery and receiving maintenance anti-tumor necrosis factor (TNF) therapy. No patients had a diverting stoma or evidence of active proctitis at the time of the procedure.

The mean time interval between fistulectomy with seton placement and the FiLaFlap procedure was 5.95 ± 1.78 months. Postoperative pain, assessed using the VAS, had a mean score of 2.20 ± 0.97, and no patients developed complications classified as Clavien-Dindo grade ≥ 2.

After a mean follow-up of 6.04 ± 0.69 months, two patients (8.3%) exhibited persistent pus or mucous secretion upon gentle finger pressure, resulting in a clinical remission rate of 91.6%. However, four patients (16.7%) showed evidence of an active residual fistula tract on endoanal ultrasound, yielding an ultrasound-confirmed remission rate of 83.3%. Among these four patients, two had Crohn’s disease and two had cryptogenic fistulas.

Regarding continence, only one patient (4.2%) reported minor incontinence (soiling) at 6 months postoperatively, while no cases of major incontinence were recorded.

Table 2 shows postoperative outcomes.

**Table 2:** Postoperative outcomes

	Patients (*n* = 24)
Mean VAS score (± SD)	2.20 ± 0.97
Clavien-Dindo complications grade > 2, *n* (%)	–
Mean follow-up, months (± SD)	6.04 ± 0.69
Clinical remission rate, *n* (%)	22 (91.6%)
Ultrasound-confirmed remission rate, *n* (%)	20 (83.3%)
Minor incontinence, *n* (%)	1 (4.2%)
Major incontinence, *n* (%)	–

## Discussion and conclusions

Complex perianal fistulas remain a significant challenge in colorectal surgery due to their high recurrence rates and the necessity of preserving sphincter function. Various sphincter-sparing techniques have been explored, among which FiLaC™ has gained popularity for its minimally invasive nature and promising outcomes. This technique utilizes a radial laser probe to deliver controlled photothermal energy, inducing fibrosis and closure of the fistula tract while minimizing damage to surrounding tissues. However, despite these advantages, FiLaC™ alone has shown variable success rates, prompting the investigation of combination strategies to improve its efficacy [7, 8].

Given these inconsistent outcomes, researchers have explored various methods to optimize closure of the internal orifice, which is a critical factor in fistula recurrence. Some studies have attempted to combine FiLaC™ with primary suturing of the internal opening, yielding mixed results. In a retrospective study by Serin et al. [9], 35 patients underwent FiLaC™ with internal orifice closure using a 2–0 polyglactin suture; at a median follow-up of 11 months, only 43% achieved complete healing, while 22% experienced persistent symptomatic drainage, suggesting that primary suturing alone may not significantly improve outcomes. Conversely, Wolicki et al. [10] reported better results in a similar cohort of 83 patients treated with FiLaC™ combined with a Z-stitch closure of the internal orifice, achieving a primary healing rate of 75% at a longer follow-up of 42 months. These discrepancies indicate that the technique used for orifice closure may play a crucial role in treatment success.

A more structured approach to improving FiLaC™ outcomes involves combining it with mucosal advancement flap closure of the internal opening. In a retrospective study by Uzun et al. [11], FiLaC™ combined with an advancement flap demonstrated a significantly higher success rate of 95.5% compared to 72% with FiLaC™ alone at a mean follow-up of 8.4 months. Similarly, Wilhelm et al. [12] analyzed a larger cohort of 117 patients with a longer median follow-up of 25.4 months, reporting a primary healing rate of 64.1% and a secondary healing rate of 88.0% after a second intervention in cases of primary failure. These findings underscore the potential of the FiLaFlap approach to enhance initial healing while also highlighting the feasibility of repeat FiLaC™ or alternative surgical strategies in cases of recurrence.

Our study adds to the limited data available in the literature, demonstrating similar findings, particularly a clinical remission rate of 91.6% and an ultrasound-confirmed remission rate of 83.3% following the FiLaFlap procedure. The technique demonstrates an excellent safety profile and was well tolerated, with minimal postoperative pain and no significant postoperative complications. Only one patient (4.2%) reported minor incontinence (soiling) at 6 months, and no cases of major incontinence were observed.

Although our follow-up duration is limited to 6 months, this timepoint is widely accepted in the literature as a reliable endpoint for evaluating fistula healing. In clinical practice, the absence of healing at 6 months is generally considered indicative of treatment failure. Moreover, most early recurrences tend to occur within this timeframe. Longer-term follow-up is ongoing and will be reported in future studies to assess durability and late recurrences.

Our results are consistent with previous studies but introduce a novel element by incorporating 3D endoanal ultrasound as an objective assessment tool. Unlike most prior studies, which relied solely on clinical evaluation, our research underscores the importance of ultrasound imaging in detecting subclinical residual tracts, which could serve as an early indicator of potential recurrence.

While pelvic MRI remains the gold standard for evaluating complex perianal fistulas, endoanal ultrasound offers several advantages [13]. It is cost-effective, can be performed in an outpatient setting with minimal patient discomfort, and, importantly, allows for continuous assessment by the same surgeon responsible for both clinical evaluation and surgery. This integrated approach enables more effective monitoring of healing progression, potentially reducing the need for additional, expensive imaging studies [14–16].

Our study has some limitations. First, the sample size is relatively small, which may limit the generalizability of our findings. Second, the retrospective nature of our analysis introduces inherent selection biases. Third, while our follow-up period provides valuable early outcome data, it may not be sufficient to capture late recurrences.

In addition, the absence of a comparative control group limits the ability to directly assess the relative efficacy of the FiLaFlap procedure against established treatments. However, considering the exploratory nature of this work and the fact that it represents one of the first systematic descriptions of this combined approach, a single-arm design was deemed appropriate to primarily evaluate feasibility, safety, and early efficacy before undertaking larger, comparative trials.

Moreover, while the inclusion of both cryptoglandular and IBD-associated fistulas may introduce some heterogeneity, it reflects real-world clinical practice and was considered appropriate in the context of a preliminary feasibility study.

Despite these limitations, the study represents an initial clinical evaluation of a novel combined approach (FiLaC™ plus mucosal advancement flap) in a real-world setting. The encouraging short-term outcomes—particularly regarding safety and ultrasound-confirmed healing—support the rationale for larger, prospective comparative studies to validate its efficacy.

Additionally, standardization of ultrasound assessment protocols and integration of imaging criteria into treatment algorithms could further refine the management of complex perianal fistulas.

In conclusion, the FiLaFlap technique may represent a valuable sphincter-preserving option for the treatment of complex perianal fistulas, demonstrating encouraging early results, namely high clinical and ultrasound-confirmed healing rates. While our results align with previous studies, further validation in larger, prospective comparative studies is essential before definitive conclusions can be drawn. Future studies should aim to compare this approach to existing techniques in order to better define its relative efficacy and indications.

## Data Availability

No datasets were generated or analyzed during the current study.
